# Propolis as an Adjunct in Non-Surgical Periodontal Therapy: Current Clinical Perspectives from a Narrative Review

**DOI:** 10.3390/jfb16070265

**Published:** 2025-07-16

**Authors:** Vitolante Pezzella, Alessandro Cuozzo, Leopoldo Mauriello, Alessandro Polizzi, Vincenzo Iorio Siciliano, Luca Ramaglia, Andrea Blasi

**Affiliations:** 1 Department of Periodontology, School of Dental Medicine, University of Naples Federico II, 80131 Naples, Italy; vitol.pezzella@studenti.unina.it (V.P.); alessandro.cuozzo@unina.it (A.C.); enzois@libero.it (V.I.S.); luca.ramaglia@unina.it (L.R.); andrea.blasi@unina.it (A.B.); 2 Department of General Surgery and Surgical-Medical Specialties, School of Dentistry, University of Catania, 95124 Catania, Italy; alessandro.polizzi@phd.unict.it

**Keywords:** periodontal disease, periodontitis, root planning, adjunctive therapy, propolis, phytotherapy

## Abstract

Non-surgical periodontal therapy (NSPT) represents the gold standard in the treatment of periodontitis, but deep periodontal pockets and complex anatomies may reduce its efficacy. Therefore, in order to enhance NSPT outcomes and reduce the need for surgical intervention, several adjunctive therapies have been proposed. Propolis, a natural substance with antimicrobial, anti-inflammatory, and healing properties, has shown promising results in controlling supragingival biofilm. This narrative review aims to assess the clinical efficacy of propolis as an adjunct to NSPT. A comprehensive search on scientific databases was conducted for randomised clinical trials (RCTs) comparing NSPT with and without propolis, or with other adjuncts or placebos. Probing depth (PD) was the primary outcome. Seven RCTs met the inclusion criteria, using different propolis formulations and application protocols. Statistically significant improvements in clinical outcomes were recorded in all analysed studies compared with NSPT alone or placebo, while benefits were less substantial compared with laser therapy and conflicting when compared with chlorhexidine. Thus propolis may be considered a promising adjunctive agent to NSPT, with the potential to improve clinical outcomes of NSPT. Nonetheless, further long-term clinical trials with larger sample size are needed to validate its clinical efficacy and to determine its adverse effects.

## 1. Introduction

Periodontitis is a chronic multifactorial inflammatory disease characterised by the progressive destruction of the tooth-supporting structures [1]. Its clinical and radiographic features are clinical attachment loss, alveolar bone loss, presence of periodontal pockets, and gingival bleeding [2]. If untreated, periodontitis may lead to the progressive destruction of periodontal supporting structures, resulting in tooth loss [3].

A bacterial biofilm deposit represents the etiological factor of periodontitis; however, its clinical manifestation and progression is strongly influenced by individual susceptibility, which is related to the host’s defences and is determined by both the regulation of inflammatory processes and the immune response [4,5,6].

Non-surgical periodontal therapy (NSPT) represents the gold standard treatment for Stage I–III periodontitis and is based on the removal of supragingival plaque and on scaling and root planing (SRP) at the sites with a periodontal probing depth ≥5 mm (subgingival instrumentation) [7], using hand instruments (i.e., curettes), sonic/ultrasonic devices, or a combination of both. Supragingival and subgingival debridement proved to be effective in reducing probing pocket depth (PD) and improving the clinical attachment level (CAL) [8]; a mean reduction of PD of 1.5 mm was estimated at 6/8 months in shallow pockets (4–6 mm), while in deeper pockets (≥7 mm) the mean PD reduction was estimated at 2.6 mm [9]. Nevertheless, NSPT alone may not always achieve the clinical endpoint of periodontal therapy (pocket closure, i.e., PD ≤ 4 mm and absence of bleeding on probing), especially in deep pockets or anatomically complex sites (root concavities, furcations), where residual biofilm may persist despite thorough instrumentation [10]. Therefore, several agents have been evaluated as adjunctive therapy to subgingival instrumentation in order to enhance clinical outcomes of NSPT and improve pocket closure, e.g., physical agents, host-modulating agents, local antimicrobials (chlorhexidine), and local and systemic antibiotics were extensively studied [2]. Recently, substances with healing effects, such as hyaluronic acid (HA) [11], and oxidising agents have also been tested [12,13,14]. However, most of these products have limitations: some do not provide additional benefits to those obtained through NSPT alone [10], others have adverse effects [15], and concerns still remain regarding cost-effectiveness, long-term safety, and antimicrobial resistance. This context has promoted increasing interest in phytotherapeutics and nutraceutical agents due to their biocompatibility, safety profile, and low cost [16,17]. Among these, propolis has emerged as one of the most promising candidates due to its broad spectrum of biological activities and favourable safety profile.

Propolis (bee glue) is a natural substance produced by honey bees, using the exudates from different plant components combined with wax and various bee enzymatic proteins [18]. Chemically, it is composed of approximately 50% resins, 30% waxes, 10% essential and aromatic oils, 5% pollen, and roughly 5% additional materials. More than 300 different chemical components have been identified, mainly including flavonoids (e.g., chrysin, pinocembrin, galangin), phenolic acids (e.g., ferulic, cinnamic, benzoic, and p-coumaric acids) and other phenolic compounds (e.g., artepillin C), esters (e.g., caffeic acid phenethyl ester (CAPE)), terpenoids, and vitamins (B1, B2, B6, C, and E) [19].

However, propolis chemical composition varies greatly depending on its geographical and botanical origin, the season of collection, and the extraction method used. Ethanol is the most common solvent employed in propolis extraction and it can significantly influence the type and concentration of the active components obtained [20].

The poplar-type propolis is the most available formulation across the world, including Europe, North America, nontropical regions of Asia, North Africa, and Oceania [21]. The basic plant sources are the bud exudates of trees of the genus Populus, chiefly the black poplar (*P. nigra*) in European propolis, and the main biologically active compounds are flavonoids and phenolic acids, while low concentrations of phenols and esters are present, which could be explained by the tendency of *Apis mellifera* to collect the bud exudates of poplars [22].

Brazilian green propolis, which originates from *Baccharis dracunculifolia*, is rich in prenylated phenylpropanoids, caffeoylquinic acids, artepillin C, and diterpenes as major compounds, while Brazilian red propolis (*Dalbergia ecastophyllum*) has isoflavonoids, neoflavonoids, pterocarpans, and lignans as predominant components.

The rich content of several bioactive molecules that act together gives propolis an important therapeutic activity and biological properties. Several studies demonstrated antibacterial, antifungal, antiviral, antiparasitic, anti-inflammatory, antiproliferative, and antioxidant effects [23,24,25,26].

Flavonoids, phenols, diterpenes, and aliphatic compounds are the main chemicals that are responsible for propolis antimicrobial activity. Propolis has a broad spectrum, that is directed both against Gram+ and Gram− bacteria (more on the former), both against aerobic and anaerobic bacteria. Antimicrobial effects are due to a direct action on the microorganism, which is expressed in the following mechanisms: increasing cell membrane permeability, reducing ATP production, disrupting of membrane potential, and decreasing bacterial mobility [19]. On the other hand, propolis also acts by stimulating the host’s immune system, through mechanisms that are not yet fully understood [27]. Bouchelaghem showed that the extracts of most types of propolis showed greater antibacterial activity against *S. aureus* and *C. albicans* [28]. Some propolis compounds can even positively modulated the antimicrobial resistance of multidrug resistant bacteria. Veiga et al. demonstrated that *Artepillin C* has a high antibacterial activity on MRSA *S. aureus* [29].

The anti-inflammatory properties of propolis are due to the content of caffeic acid phenyl ester (CAPE), which stops the arachidonic acid pathway by inhibiting the activity of cyclooxygenase (COX) and lipoxygenase (LOX). As a consequence, a reduction in the production of prostaglandins and leukotrienes, involved in phlogistic mechanisms is recorded [30]. CAPE also inhibits NF-kappa B activation [31], enhancing the production of anti-inflammatory cytokines IL4 and IL10 and decreasing monocyte and neutrophil infiltrate [32]. Terpenoids extracted from propolis reduce the expression of inflammatory mediators, such as nitric oxide synthase (NOS) [33], while phenolics and flavonoids have strong antioxidant activities.

Moreover, several studies showed that propolis can inhibit proliferation, angiogenesis, and metastasis of cancer cells and stimulate apoptosis [34].

As a result, an increasing use of propolis has also been observed in dentistry. Currently it can be applied in the dental field with different delivery formulations, achieving good results in ulcer healing, prevention of caries, as a cavity disinfecting agent, and even as a direct pulp capping material [35,36,37,38]. Therefore, propolis has proven useful in the treatment of gingivitis, as it reduces plaque accumulation and improves inflammatory indices [39]. It is effective in controlling supragingival plaque through the inhibition of bacterial adhesion and metabolism, by promoting calcium phosphate deposition on tooth surfaces, aiding in plaque prevention, and through antimicrobial effects against different oral pathogens [40]. Gebara et al. showed that propolis is effective in vitro against several periodontopathogenic bacteria (such as *Prevotella intermedia*, *Porphyromoras gingivalis*, *Actinobacillus actinomycetemcomitans*, *Fusobacterium nucleatum*) [41], while Yoshimasu et al. demonstrated a bactericidal action against *Phorpyromonas gingivalis*, where artepillin C had bacteriostatic activity and ursolic acid showed bactericidal activity [42].

In recent years, some studies have explored the use of propolis in the treatment of periodontitis, both as an adjunct to SRP and as an additional therapy to periodontal surgery.

Given its wide-ranging bioactive profile and the limitations of conventional adjunctive therapies, propolis represents an interesting topic of study in periodontal therapy.

Furthermore, the high incidence of periodontitis and its established associations with systemic conditions, such as diabetes and cardiovascular diseases, suggest that the investigation of propolis in this context is of particular interest for both clinical dentistry and public health.

This narrative review aims to critically analyse clinical application-related research on the effects of propolis as an adjunct to NSPT based on PubMed, Scopus, and Web of Science databases up to April 2025.

## 2. Materials and Methods

The aim of the present review was to determine the clinical efficacy of propolis as an adjunctive agent to NSPT compared with scaling and root planing alone or other adjunctive agents. The primary outcome of this study was PD, while the secondary outcome was CAL.

An electronic literature search was conducted using Scopus, PubMed, and Web of Science databases up to and including April 2025. A combination of keywords, including “propolis”, “periodontitis”, “adjunctive therapy”, and “periodontal disease” were used in the databases following their syntax rules. All combinations using (AND, OR) were utilised to refine the search results.

Studies were included in the literature review in accordance with the following inclusion criteria: (1) studies focused on clinical effects of propolis as an adjunct to NSPT; (2) studies performed in vivo on humans; (3) randomised controlled trials (RCTs). 

Studies were excluded from the review in accordance with the following exclusion criteria: (1) studies including patients with systemic conditions; (2) studies that did not use propolis as an adjuvant to NSPT or used it to treat other oral conditions; (3) studies using propolis only in combination with other adjunctive therapies; (4) studies using propolis without SRP; (5) studies not available in English.

All retrieved studies from the electronic literature search were imported into a reference management web app (e.g., Rayyan) and duplicates were removed. Two reviewers (V.P. and L.M.) independently screened titles and abstracts of all studies during the preliminary round of study selection to assess eligibility based on predefined inclusion and exclusion criteria. The full texts of the studies that met the inclusion criteria were gathered. Further screening of the full texts of the selected articles was performed during the second round of the study selection, and articles that did not match the inclusion criteria were excluded from consideration. In the presence of disagreement between reviewers, the decision regarding study eligibility was attempted by reviewers reaching a consensus. Where continued disagreement was apparent, an arbitrator (A.C.) judged the study inclusion.

The following data were extracted from each eligible trial: (a) general study characteristics: first author name, year of publication, country, study design, setting, number of patients; (b) patient characteristics: male/female ratio, mean age of patients, smoking habits; (c) type of periodontitis; (d) treatment: details of the adjunctive therapies, placebo and control groups; (f) outcomes: PD and CAL at different follow-up timepoints.

**Figure 1:**
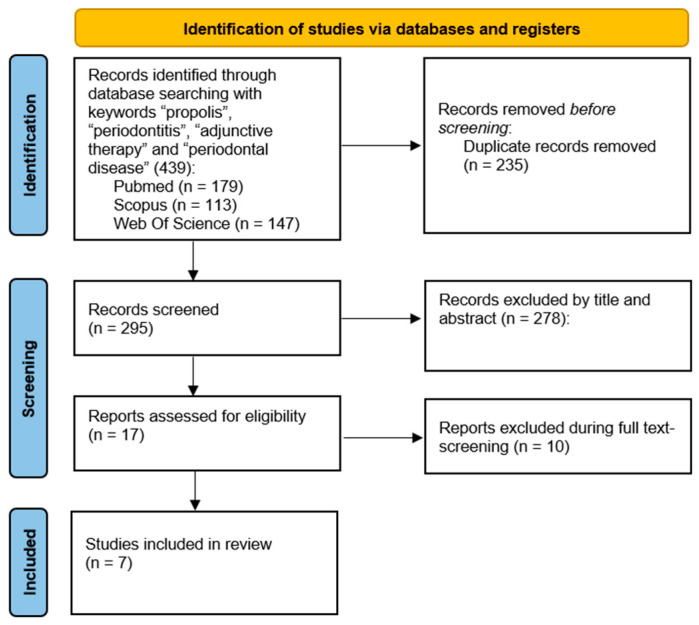
Flowchart article retrieval process.

The article selection process is illustrated in Figure 1.

A comparative narrative analysis was performed by examining differences and similarities across studies, focusing on their key characteristics and outcomes. The main findings were summarised in comparative tables and discussed accordingly.

## 3. Results

Based on the established inclusion criteria, seven relevant RCTs investigating the clinical effects of propolis as an adjunct to NSPT in patients diagnosed with periodontitis were identified. The main characteristics and clinical findings are presented in Table 1.

All studies excluded patients with a smoking habit. Six out of seven included studies reported statistically significant clinical improvements in the primary outcome (PD) following the use of propolis as an adjunct to NSPT.

Six trials have investigated the potential beneficial effects of the topical application of propolis directly in periodontal pockets after SRP. In three trials, the formulation analysed was a propolis solution for subgingival irrigation, which was compared with SRP alone or a placebo (saline solution). Statistically significative differences were found by de Andrade et al., who assessed the effects of a 20% hydroalcoholic solution of propolis extract, recording a PD reduction of 1.50 ± 1.40 mm and 1.30 ± 1.41 mm in test and control groups, respectively, at three-month follow-up [43]. Pundir et al. used a 20% propolis hydroalcoholic solution, applied 24 h after SRP, within a one-stage full mouth disinfection (OSFMD) protocol [44]. This included brushing the dorsum of the tongue for 60 s with the solution, rinsing the mouth twice with the same solution for 1 min, and subgingival irrigation of periodontal pockets. Statistically significant improvements were observed in all clinical indices (PI, GI, BoP, PD, and CAL) between the test and control groups at 12-week follow-up. In particular, PD was reduced from 5.87 ± 0.92 mm to 3.87 ± 0.92 mm in the test group, and from 5.53 ± 0.52 mm to 4.53 ± 0.52 mm in the control group. Sahu et al. delivered a subgingival formulation of propolis nanoparticle solution and used isoamyl-2-cyanoacrylate for sealing periodontal pockets [45]. All clinical parameters (BoP, PD, CAL) showed significant improvement within the test group at the end of the 3-month follow-up: PD median values were 2.45 mm for the test group and 3.15 mm for the control group, while PD median value at baseline was 4.90 mm for both groups.

Seth et al. evaluated subgingival irrigation with 25% propolis extract compared with irrigation with 0.2% chlorhexidine [46]. After one month, a reduction in PD of 34.68% was observed in the propolis group (from 6.20 ± 1.00 mm to 4.10 ± 1.16 mm), and 43.84% in the chlorhexidine group (from 6.50 ± 1.05 mm to 3.65 ± 1.18 mm). Although chlorhexidine showed slightly better results, no statistically significant differences were found between the two groups in terms of PD.

**Table 1:** Characteristics and outcomes of all included randomised clinical trials of this review.

Authors (Year)—Country	Study Design	Setting	N. of Patients	Gender (M/F)	Mean Age	Type of Periodontitis	Treatment	Results (PD—CAL)	Follow-Up
Sanghani N. et al. (2014)—India [47]	RCT	University	20	9 M/11 F	35.60 ± 12.20	Chronic Periodontitis	Test Group: SRP + subgingival administration of propolis (~5 mg) Control Group: SRP	Test Group PD Baseline: 5.35 ± 0.67 15 days: 4.60 ± 0.68 1 month: 3.60 ± 0.68 CAL Baseline: 3.35 ± 0.67 15 days: 2.10 ± 0.79 1 month: 1.60 ± 0.68 Control Group PD Baseline: 5.10 ± 0.55 15 days: 4.55 ± 0.83 1 month: 3.75 ± 0.79 CAL Baseline: 3.10 ± 0.55 15 days: 2.55 ± 0.83 1 month: 1.75 ± 0.79	15 days 1 month
De Andrade D.P. et al. (2017)—Brazil [43]	RCT	University	16	10 M/6 F	Test group: 50.22 ± 7.75 Control group: 48.00 ± 9.10	Chronic Periodontitis	Test Group: SRP + subgingival irrigation with a hydroalcoholic solution of propolis extract 20%; second irrigation after 15 days Control Group: SRP + subgingival irrigation with a saline solution; second irrigation after 15 days	Test Group PD change Baseline–45 days: 1.42 ± 1.37 Baseline–75 days: 1.48 ± 1.39 Baseline–90 days: 1.50 ± 1.40 45 days–75 days: 0.06 ± 0.72 45 days–90 days: 0.08 ± 0.78 75 days–90 days: 0.02 ± 0.55 Control Group PD change Baseline–45 days: 1.13 ± 1.29 Baseline–75 days: 1.23 ± 1.34 Baseline–90 days: 1.30 ± 1.41 45 days–75 days: 0.09 ± 0.69 45 days–90 days: 0.16 ± 0.83 75 days–90 days: 0.07 ± 0.57	45 days 75 days 90 days
Pundir A. et al. (2017)—India [44]	RCT	University	30	N.A.	25–55	Chronic Periodontitis	Test Group: SRP + one-stage full mouth disinfection (brushing the dorsum of the tongue for 60 s + rinsing the mouth twice for 1 min with propolis solution + subgingival irrigation with 20% propolis hydroalcoholic solution) 24 h after SRP Control Group: SRP	Test Group PD Baseline: 5.87 ± 0.92 4 weeks: 3.87 ± 0.92 12 weeks: 3.87 ± 0.92 CAL Baseline: 3.87 ± 0.92 4 weeks: 1.47 ± 1.51 12 weeks: 1.47 ± 1.51 Control Group PD Baseline: 5.53 ± 0.52 4 weeks: 4.53 ± 0.52 12 weeks: 4.53 ± 0.52 CAL Baseline: 3.53 ± 0.52 4 weeks: 2.53 ± 0.52 12 weeks: 2.53 ± 0.52	4 weeks 12 weeks
Seth T. et al. (2022)—India [46]	RCT	University	20	N.A.	18–55	Mild/Moderate Periodontitis	Test Group: Single-phase full-mouth SRP + subgingival irrigation for 30 s for 5 mL with 25% propolis extract. Subgingival irrigation repeated on the 7th and 15th day from the day of first application Control Group: Single-phase full-mouth SRP + subgingival irrigation for 30 s for 5 mL with 0.2% chlorhexidine. Subgingival irrigation repeated on the 7th and15th day from the day of first application	Test Group PD Baseline: 6.20 ± 1.00 15 days: 5.05 ± 1.14 30 days: 4.10 ± 1.16 Control Group PD Baseline 6.50 ± 1.05 15 days: 4.95 ± 1.09 30 days: 3.65 ± 1.18	15 days 30 days
Sahu S. et al. (2023)—India [45]	RCT	University	40	30 M/10 F	Test group: 49.90 ± 11.60 Control group: 50.10 ± 12.20	Generalised Stage II-III	Test Group: SRP + subgingival administration of propolis nanoparticle solution + pocket sealing with cyanoacrylate Control Group: SRP + subgingival administration of saline solution + pocket sealing with cyanoacrylate	Test Group * PD Baseline: 4.90 (4.00–6.65) 1 month: 2.90 (2.00–4.65) 3 months: 2.45 (2.00–3.20) CAL Baseline: 9.65 (8.95–11.30) 1 month: 7.65 (6.55–9.95) 3 months: 7.20 (6.75–9.95) Control Group * PD Baseline: 4.90 (4.00–6.00) 1 month: 3.30 (3.00–5.00) 3 months: 3.15 (2.00–3.00) CAL Baseline: 9.55 (8.00–11.55) 1 month: 7.95 (6.00–10.00) 3 months: 7.80 (6.00–9.00)	1 month 3 months
Aggarwal R. et al. (2023)—India [48]	RCT	University	30	N.A.	N.A.	Moderate/Severe Chronic Periodontitis	Test group 1: SRP + application in periodontal pocket of propolis gel Test group 2: SRP + diode laser (set at 1.5 W, 940 nm, 30 s, continuous wave) into the periodontal pocket Control group: SRP	Test Group 1 PD 1 month: 4.22 ± 0.59 3 months: 3.93 ± 0.63 CAL 1 month: 1.92 ± 0.40 3 months: 1.67 ± 0.40 Test Group 2 PD 1 month: 4.04 ± 0.48 3 months: 2.77 ± 0.60 CAL 1 month: 0.97 ± 0.11 3 months: 1.26 ± 0.2 Control Group PD 1 month: 4.40 ± 0.54 3 months: 3.58 ± 0.53 CAL 1 month: 1.43 ± 0.29 3 months: 1.12 ± 0.50	1 month 3 months
Waqar et al. (2024)—Pakistan [49]	RCT	University	100	0 M/100 F	N.A.	Chronic Periodontitis (Stage I–II)	Test Group: SRP + 20% propolis mouthwash twice daily for six weeks Control Group: SRP + 0.2% chlorhexidine mouthwash twice daily for six weeks	Test Group PD Baseline: 4.67 (4.56–4.89) 6 weeks: 4.01 (3.72–4.15) 12 weeks: 3.59 (3.28–3.92) CAL Baseline: 4.45 ± 0.73 6 weeks: 4.15 ± 0.57 12 weeks: 3.77 ± 0.51 Control Group PD Baseline: 4.65 (4.43–4.89) 6 weeks: 4.44 (4.16–4.63) 12 weeks: 4.25 (4.02–4.48) CAL Baseline: 4.80 ± 0.70 6 weeks: 4.50 ± 0.61 12 weeks: 4.19 ± 0.56	6 weeks 12 weeks

* Value expressed as median (range).

Two other trials used propolis in different topical formulations. Sanghani et al. compared a subgingival application of about 5 mg of Indian propolis after SRP (test group) to SRP alone (control group), recording statistically significant improvements in all clinical parameters at one-month follow-up (PD test: 3.60 ± 0.68 mm; PD control: 3.75 ± 0.79 mm) [47]. Aggarwal et al. investigated the clinical effects of propolis (propolis extract gel) or diode laser as adjuncts to SRP (test groups) compared with SRP alone (control group). No significant differences were found in clinical parameters at 3-month follow-up in the propolis gel group (PD: 3.93 ± 0.63 mm), while diode laser as an adjunct to SRP was highly effective in reducing gingival inflammation, probing depth, and clinical attachment level (PD: 2.77 ± 0.60 mm) [48].

Finally, Waqar et al. administered a propolis mouthwash following SRP (20% propolis mouthwash twice daily for six weeks) [49]. The control group received a chlorhexidine mouthwash (0.2% chlorhexidine mouthwash twice daily for six weeks). After 12 weeks, improvements in clinical parameters were recorded in both in the test group (PD reduced from 4.67 mm to 3.59 mm) and the control group (PD reduced from 4.65 mm to 4.25 mm); however, propolis was significantly more effective in improving BoP (77.20 at baseline; 14.60 at 12-week follow-up) than chlorhexidine (77.30 at baseline; 22.80 at 12-week follow-up).

Only one study reported adverse effects in a patient treated with propolis formulations. de Andrade et al. found lesions similar to ulcers or burns in the sites that received a subgingival irrigation with a hydroalcoholic solution of propolis extract [43]. According to the authors, these lesions should be attributed to the presence of caffeic acid in the solution.

## 4. Discussion

**Figure 2:**
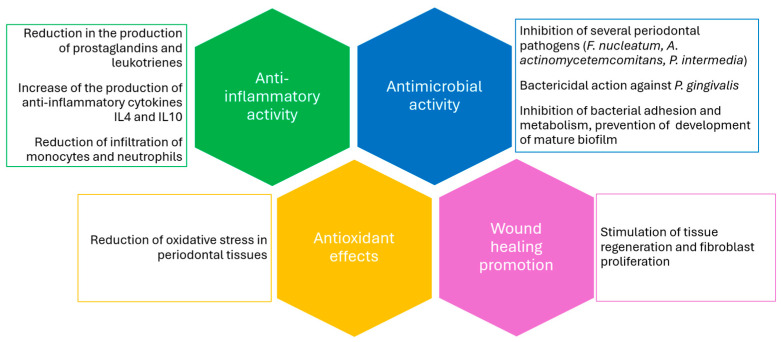
Mechanism of action of propolis on periodontitis.

In recent years, propolis has gained increasing attention as a natural adjunct to NSPT. Its use seems to enhance the clinical outcomes of SRP, particularly by improving PD reduction and increasing the percentage of pocket closure during the re-evaluation phase of periodontal therapy, potentially reducing the need for surgical intervention. Propolis exhibits several biological properties that support its application in this context, including antimicrobial activity against the main periodontopathogenic bacteria [41,42], antioxidant effects that may counteract oxidative stress in chronic inflammation [50], anti-inflammatory action that could help control the inflammatory response of the periodontal tissue, and potential wound-healing benefits [30,32] (Figure 2).

This narrative review examined the latest clinical evidence evaluating the effectiveness of propolis as an adjunct to NSPT.

Four of the seven analysed studies directly compared propolis with NSPT alone or with a placebo. All four reported statistically significant improvements in clinical parameters, particularly in terms of PD reduction and CAL gain. However, the interpretation and comparison of these results is limited by several methodological differences. First, the results were expressed with different statistical measures. Sahu et al. [45] presented data as medians and ranges, while the other studies reported clinical outcomes as mean and standard deviation. de Andrade et al. [43] focused solely on PD reduction, while Sanghani et al. [47], as well as Pundir et al. [44], evaluated both the PD and the CAL, providing baseline and follow-up values, for both test and control groups. Additionally, variations in the formulations of propolis and their application protocols further limited the comparability of findings. de Andrade et al. [43] and Pundir et al. [44] used a hydroalcoholic solution of propolis extract 20%. In the former study, test sites received subgingival irrigation, while, in the latter, propolis was also used for brushing the tongue and rinsing the mouth (OSFMD protocol). de Andrade et al. [43] reported a PD reduction of 1.50 mm at 3-month follow-up, while Pundir et al. [44] obtained about 2 mm, therefore suggesting that an extended use of propolis to the whole oral cavity may be more effective than its use in a single pocket.

Seth et al. treated test sites using subgingival irrigation with 25% propolis extract, applied after SRP and then repeated on the 7th day and 15th day from the day of first application, reporting a PD reduction of 2.10 mm at only 1-month follow-up. These results suggest that a higher concentration, administered repeatedly, may accelerate periodontal healing [46].

Waqar et al., using 20% propolis mouthwash twice daily for six weeks, reported a PD reduction of approximately 1.39 mm [49]. These findings reinforce the idea that topical application of propolis in periodontal pockets may provide superior benefits compared with mouthwash after SRP.

The reported reductions in PD and gains in CAL are generally considered clinically meaningful in periodontal therapy (≥1 mm for PD, ≥0.5 mm for CAL), indicating improvements in pocket health and tissue stability and a lower risk of disease progression [51].

Furthermore, these last two studies compared the efficacy of propolis with chlorhexidine as an adjunct to NSPT. Seth et al. [46] showed slightly better results in the group that received irrigation with chlorhexidine, finding no statistically significant difference. Conversely, Waqar et al. [49] found better results in the group receiving the propolis mouthwash compared with the group treated with the chlorhexidine mouthwash, recording statistically significant differences for all clinical parameters (PD, CAL, BoP). However, although the study had the largest sample size (n = 100), the presence of only female individuals constituted an important bias.

Aggarwal et al. compared propolis to laser therapy, finding significative better results in the sites treated with laser in both PD reduction and CAL gain [48].

Compared with conventional agents such as chlorhexidine, propolis presents several potential advantages, e.g., chlorhexidine is effective against a wide range of oral pathogens but its prolonged use is associated with side effects including tooth staining, altered taste sensation, and potential cytotoxicity to oral tissues [52]. In contrast, propolis demonstrates antimicrobial efficacy with a lower risk of inducing resistance and it is more biocompatible [27]. Moreover, propolis also exhibits anti-inflammatory, antioxidant, and wound-healing properties, which could contribute to improved clinical outcomes in periodontitis without the drawbacks of synthetic chemical agents.

The heterogeneity of the included studies represents a limitation of this review. The analysed trials varied considerably in terms of propolis formulation, delivery protocols, and classification systems used for diagnosing periodontitis. Follow-up durations were often short, ranging from 1 to 3 months, which limits the ability to evaluate the long-term clinical benefits and stability of results. Moreover, most studies involved small sample sizes and were conducted in single-centre settings, with a lack of multicentre or large-scale RCTs. Finally, the absence of a bias assessment tool in this review further limits the strength of the conclusions.

Adverse effects were recorded only in one trial; however, the recorded effects (ulcers) may not be related to propolis. Therefore, more pharmacological studies are needed to understand propolis effects on the human body since it may represent a limitation to its use.

Although the results as an adjunct to NSPT are positive and promising, propolis clinical benefits remain difficult to predict due to the lack of well-defined application protocols and the availability of several commercial formulations, none of which have been extensively validated through large-scale clinical trials.

Further research should include multicentre RCTs with larger sample sizes and longer follow-up periods to assess the long-term efficacy of propolis as an adjunct to NSPT. The standardisation of formulations and delivery protocols is also needed, as well as studies investigating its mechanisms of action in periodontal tissues. Future investigations could also explore the synergistic combination of propolis with other therapeutic agents to enhance clinical outcomes.

## 5. Conclusions

Despite the limitations of this study, propolis represents a promising adjunctive agent to NSPT, with the potential to improve clinical outcomes such as probing depth reduction and clinical attachment gain.

However, further long-term clinical trials with larger sample size are needed to validate clinical efficacy and to establish optimal clinical protocols in periodontal treatment.

## Data Availability

The original contributions presented in this study are included in the article; further inquiries can be directed to the corresponding author.

## Abbreviations

The following abbreviations are used in this manuscript:

NSPT	Non-Surgical Periodontal Therapy
RCT	Randomised Controlled Trial
SRP	Scaling and Root Planing
PD	Probing Depth
CAL	Clinical Attachment Level
BoP	Bleeding on Probing
OSFMD	One-Stage Full Mouth Disinfection
GI	Gingival Index
PI	Plaque Index

## References

[1] Papapanou P.N., Sanz M., Buduneli N., Dietrich T., Feres M., Fine D.H., Flemmig T.F., Garcia R., Giannobile W.V., Graziani F. (2018). Periodontitis: Consensus Report of Workgroup 2 of the 2017 World Workshop on the Classification of Periodontal and Peri-Implant Diseases and Conditions. J. Periodontol..

[2] Sanz M., Herrera D., Kebschull M., Chapple I., Jepsen S., Beglundh T., Sculean A., Tonetti M.S., Merete Aass A., Aimetti M. (2020). Treatment of Stage I–III Periodontitis—The EFP S3 Level Clinical Practice Guideline. J. Clin. Periodontol..

[3] Kassebaum N.J., Bernabé E., Dahiya M., Bhandari B., Murray C.J.L., Marcenes W. (2014). Global Burden of Severe Tooth Loss: A Systematic Review and Meta-Analysis. J. Dent. Res..

[4] Raittio E., Leite F.R.M., Machado V., Botelho J., Nascimento G.G. (2024). Do All Individuals Benefit Equally from Non-Surgical Periodontal Therapy? Secondary Analyses of Systematic Review Data. J. Periodontal Res..

[5] Herrera D., Sanz M., Shapira L., Brotons C., Chapple I., Frese T., Graziani F., Hobbs F.D.R., Huck O., Hummers E. (2023). Association between Periodontal Diseases and Cardiovascular Diseases, Diabetes and Respiratory Diseases: Consensus Report of the Joint Workshop by the European Federation of Periodontology (EFP) and the European Arm of the World Organization of Family Doctors (WONCA Europe). J. Clin. Periodontol..

[6] Isola G., Tartaglia G.M., Santonocito S., Polizzi A., Williams R.C., Iorio-Siciliano V. (2023). Impact of N-Terminal pro-B-Type Natriuretic Peptide and Related Inflammatory Biomarkers on Periodontal Treatment Outcomes in Patients with Periodontitis: An Explorative Human Randomized-Controlled Clinical Trial. J. Periodontol..

[7] Kwon T.H., Lamster I.B., Levin L. (2021). Current Concepts in the Management of Periodontitis. Int. Dent. J..

[8] Van der Weijden G.A., Dekkers G.J., Slot D.E. (2019). Success of Non-Surgical Periodontal Therapy in Adult Periodontitis Patients: A Retrospective Analysis. Int. J. Dent. Hyg..

[9] Suvan J., Leira Y., Moreno Sancho F.M., Graziani F., Derks J., Tomasi C. (2020). Subgingival Instrumentation for Treatment of Periodontitis. A Systematic Review. J. Clin. Periodontol..

[10] Haas A.N., Furlaneto F., Gaio E.J., Gomes S.C., Palioto D.B., Castilho R.M., Sanz M., Messora M.R. (2021). New Tendencies in Non-Surgical Periodontal Therapy. Braz. Oral Res..

[11] Iorio-Siciliano V., Blasi A., Mauriello L., Salvi G.E., Ramaglia L., Sculean A. (2025). Non-Surgical Treatment of Moderate Periodontal Intrabony Defects With Adjunctive Cross-Linked Hyaluronic Acid: A Single-Blinded Randomized Controlled Clinical Trial. J. Clin. Periodontol..

[12] Kardaras G., Marcovici I., Rusu D., Dehelean C., Coricovac D., Iorio-Siciliano V., Sculean A., Stratul S.-I. (2023). In-Vitro Safety Evaluation of Sodium Hypochlorite (NaOCl) as Part of Step 2 and Maintenance Therapy Protocols in Patients with Periodontitis Stages III-IV. Oral Health Prev. Dent..

[13] Radulescu V., Boariu M.I., Rusu D., Roman A., Surlin P., Voicu A., Didilescu A.C., Jentsch H., Siciliano V.I., Ramaglia L. (2022). Clinical and Microbiological Effects of a Single Application of Sodium Hypochlorite Gel during Subgingival Re-Instrumentation: A Triple-Blind Randomized Placebo-Controlled Clinical Trial. Clin. Oral Investig..

[14] Iorio-Siciliano V., Ramaglia L., Isola G., Blasi A., Salvi G.E., Sculean A. (2021). Changes in Clinical Parameters Following Adjunctive Local Sodium Hypochlorite Gel in Minimally Invasive Nonsurgical Therapy (MINST) of Periodontal Pockets: A 6-Month Randomized Controlled Clinical Trial. Clin. Oral Investig..

[15] Wei Y., Deng Y., Ma S., Ran M., Jia Y., Meng J., Han F., Gou J., Yin T., He H. (2021). Local Drug Delivery Systems as Therapeutic Strategies against Periodontitis: A Systematic Review. J. Control. Release.

[16] Isola G., Polizzi A., Iorio-Siciliano V., Alibrandi A., Ramaglia L., Leonardi R. (2021). Effectiveness of a Nutraceutical Agent in the Non-Surgical Periodontal Therapy: A Randomized, Controlled Clinical Trial. Clin. Oral Investig..

[17] Gawish A.S., ElMofty M.S., Jambi S., Felemban D., Ragheb Y.S.E., Elsayed S.A. (2024). Phytotherapy in Periodontics as an Effective and Sustainable Supplemental Treatment: A Narrative Review. J. Periodontal Implant. Sci..

[18] Martinotti S., Bonsignore G., Ranzato E. (2025). Propolis: A Natural Substance with Multifaceted Properties and Activities. Int. J. Mol. Sci..

[19] Przybyłek I., Karpiński T.M. (2019). Antibacterial Properties of Propolis. Molecules.

[20] El-Sakhawy M., Salama A., Tohamy H.A.S. (2024). Applications of Propolis-Based Materials in Wound Healing. Arch. Dermatol. Res..

[21] Jiang X.-S., Xie H.-Q., Li C.-G., You M.-M., Zheng Y.-F., Li G.Q., Chen X., Zhang C.-P., Hu F.-L. (2020). Chinese Propolis Inhibits the Proliferation of Human Gastric Cancer Cells by Inducing Apoptosis and Cell Cycle Arrest. Evid. Based Complement. Altern. Med..

[22] Wieczorek P.P., Hudz N., Yezerska O., Horčinová-Sedláčková V., Shanaida M., Korytniuk O., Jasicka-Misiak I. (2022). Chemical Variability and Pharmacological Potential of Propolis as a Source for the Development of New Pharmaceutical Products. Molecules.

[23] Mirzoeva O.K., Calder P.C. (1996). The Effect of Propolis and Its Components on Eicosanoid Production during the Inflammatory Response. Prostaglandins Leukot. Essent. Fatty Acids.

[24] Martinello M., Mutinelli F. (2021). Antioxidant Activity in Bee Products: A Review. Antioxidants.

[25] Franchin M., Cólon D.F., Castanheira F.V.S., da Cunha M.G., Bueno-Silva B., Alencar S.M., Cunha T.M., Rosalen P.L. (2016). Vestitol Isolated from Brazilian Red Propolis Inhibits Neutrophils Migration in the Inflammatory Process: Elucidation of the Mechanism of Action. J. Nat. Prod..

[26] Forma E., Bryś M. (2021). Anticancer Activity of Propolis and Its Compounds. Nutrients.

[27] Almuhayawi M.S. (2020). Propolis as a Novel Antibacterial Agent. Saudi J. Biol. Sci..

[28] Bouchelaghem S. (2022). Propolis Characterization and Antimicrobial Activities against Staphylococcus Aureus and Candida Albicans: A Review. Saudi J. Biol. Sci..

[29] Veiga R.S., De Mendonça S., Mendes P.B., Paulino N., Mimica M.J., Lagareiro Netto A.A., Lira I.S., López B.G.-C., Negrão V., Marcucci M.C. (2017). Artepillin C and Phenolic Compounds Responsible for Antimicrobial and Antioxidant Activity of Green Propolis and *Baccharis dracunculifolia* DC. J. Appl. Microbiol..

[30] Khurshid Z., Naseem M., Zafar M.S., Najeeb S., Zohaib S. (2017). Propolis: A Natural Biomaterial for Dental and Oral Healthcare. J. Dent. Res. Dent. Clin. Dent. Prospect..

[31] Natarajan K., Singh S., Burke T.R., Grunberger D., Aggarwal B.B. (1996). Caffeic Acid Phenethyl Ester Is a Potent and Specific Inhibitor of Activation of Nuclear Transcription Factor NF-Kappa B. Proc. Natl. Acad. Sci. USA.

[32] Orban Z., Mitsiades N., Burke T.R., Tsokos M., Chrousos G.P. (2000). Caffeic Acid Phenethyl Ester Induces Leukocyte Apoptosis, Modulates Nuclear Factor-Kappa B and Suppresses Acute Inflammation. Neuroimmunomodulation.

[33] Zhang W., Cai Y., Chen X., Ji T., Sun L. (2020). Optimized Extraction Based on the Terpenoids of *Heterotrigona Itama* Propolis and Their Antioxidative and Anti-inflammatory Activities. J. Food Biochem..

[34] Elumalai P., Muninathan N., Megalatha S.T., Suresh A., Kumar K.S., Jhansi N., Kalaivani K., Krishnamoorthy G. (2022). An Insight into Anticancer Effect of Propolis and Its Constituents: A Review of Molecular Mechanisms. Evid.-Based Complement. Altern. Med..

[35] Rodrigues Neto E.M., Valadas L.A.R., Lobo P.L.D., Fonseca S.G.D.C., Fechine F.V., Lotif M.A.L., Bandeira M.A.M., Mendonça J.F., de Mendonça K.M., Fonteles M.M.D.F. (2021). Antimicrobial Efficacy of Propolis-Containing Varnish in Children: A Randomized and Double-Blind Clinical Trial. Evid.-Based Complement. Altern. Med..

[36] Ahangari Z., Naseri M., Jalili M., Mansouri Y., Mashhadiabbas F., Torkaman A. (2012). Effect of Propolis on Dentin Regeneration and the Potential Role of Dental Pulp Stem Cell in Guinea Pigs. Cell J..

[37] Alghutaimel H. (2024). Endodontic Applications of Propolis in Primary and Permanent Teeth: A Scoping Review of Clinical Studies. Eur. Endod. J..

[38] Samet N., Laurent C., Susarla S.M., Samet-Rubinsteen N. (2007). The Effect of Bee Propolis on Recurrent Aphthous Stomatitis: A Pilot Study. Clin. Oral Investig..

[39] Khabazian A., Mirhashemi F.S., Sadeghi F. (2025). Investigating the Effect of Propolis-Containing Chewing Gum in Comparison with Propolis-Containing Mouthwash on Reducing Gingival Inflammation in Patients with Gingivitis. BMC Oral Health.

[40] Ballouk M.A.-H., Altinawi M., Al-Kafri A., Zeitounlouian T.S., Fudalej P.S. (2025). Propolis Mouthwashes Efficacy in Managing Gingivitis and Periodontitis: A Systematic Review of the Latest Findings. BDJ Open.

[41] Gebara E.C.E., Lima L.A., Mayer M.P.A. (2002). Propolis Antimicrobial Activity against Periodontopathic Bacteria. Braz. J. Microbiol..

[42] Yoshimasu Y., Ikeda T., Sakai N., Yagi A., Hirayama S., Morinaga Y., Furukawa S., Nakao R. (2018). Rapid Bactericidal Action of Propolis against *Porphyromonas Gingivalis*. J. Dent. Res..

[43] de Andrade D.P., Carvanho I.C., Gadoi B.H., Rosa L.C.L., Barreto L.M.R.C., Pallos D. (2017). Subgingival Irrigation with a Solution of 20% Propolis Extract as an Adjunct to Non-Surgical Periodontal Treatment: A Preliminary Study. J. Int. Acad. Periodontol..

[44] Pundir A., Vishwanath A., Pundir S., Swati M., Banchhor S., Jabee S. (2017). One-Stage Full Mouth Disinfection Using 20% Propolis Hydroalcoholic Solution: A Clinico-Microbiologic Study. Contemp. Clin. Dent..

[45] Sahu S.A., Panda S., Das A.C., Mishra L., Rath S., Sokolowski K., Kumar M., Mohanty R., Nayak R., Satpathy A. (2023). Efficacy of Sub-Gingivally Delivered Propolis Nanoparticle in Non-Surgical Management of Periodontal Pocket: A Randomized Clinical Trial. Biomolecules.

[46] Seth T., Kale T., Lendhey S., Bhalerao P. (2022). Comparative Evaluation of Subgingival Irrigation with Propolis Extract versus Chlorhexidine as an Adjunct to Scaling and Root Planing for the Treatment of Chronic Periodontitis: A Randomized Controlled Trial. J. Indian Soc. Periodontol..

[47] Sanghani N.N., Shivaprasad B.M., Savita S. (2014). Health from the Hive: Propolis as an Adjuvant in the Treatment of Chronic Periodontitis—A Clinicomicrobiologic Study. J. Clin. Diagn. Res..

[48] Aggarwal R., Bawa S.S., Palwankar P., Kaur S., Choudhary D., Kochar D. (2023). To Evaluate the Clinical Efficacy of 940 Nm Diode Laser and Propolis Gel (A Natural Product) in Adjunct to Scaling and Root Planing in Treatment of Chronic Periodontitis. J. Pharm. Bioallied Sci..

[49] Waqar S.M., Razi A., Qureshi S.S., Saher F., Zaidi S.J.A., Kumar C. (2024). Comparative Evaluation of Propolis Mouthwash with 0.2% Chlorhexidine Mouthwash as an Adjunct to Mechanical Therapy in Improving the Periodontitis among Perimenopausal Women: A Randomized Controlled Trial. BMC Oral Health.

[50] Kocot J., Kiełczykowska M., Luchowska-Kocot D., Kurzepa J., Musik I. (2018). Antioxidant Potential of Propolis, Bee Pollen, and Royal Jelly: Possible Medical Application. Oxid. Med. Cell Longev..

[51] Greenstein G. (2003). Clinical versus Statistical Significance as They Relate to the Efficacy of Periodontal Therapy. J. Am. Dent. Assoc..

[52] Poppolo Deus F., Ouanounou A. (2022). Chlorhexidine in Dentistry: Pharmacology, Uses, and Adverse Effects. Int. Dent. J..

